# Traditional Yellow Dyes Used in the 21st Century in Central Iran: The Knowledge of Master Dyers Revealed by HPLC-DAD and UHPLC-HRMS/MS

**DOI:** 10.3390/molecules25040908

**Published:** 2020-02-18

**Authors:** Samaneh Sharif, Paula Nabais, Maria J. Melo, M. Conceição Oliveira

**Affiliations:** 1 LAQV-REQUIMTE and Department of Conservation and Restoration, NOVA School of Sciences and Technology of NOVA University Lisbon, 2829-516 Monte da Caparica, Portugal; s.sharif@campus.fct.unl.pt (S.S.); p.nabais@campus.fct.unl.pt (P.N.); 2 IEM, Campus de Campolide, NOVA University Lisbon, 1070-312 Lisboa, Portugal; 3 Centro de Química Estrutural, Instituto Superior Técnico, Universidade de Lisboa, 1049-001 Lisboa, Portugal

**Keywords:** dye analysis, soft extraction methods, persian dyes, flavonoids, HPLC, mass spectrometry, yellow colors, conservation

## Abstract

This work provides new knowledge on natural yellows used in Iran. Seven biological sources were selected based on interviews with dye masters in Isfahan workshops (Iran). *Delphinium semibarbatum*, *Eremostachys laevigata*, *Prangos ferulacea*, *Morus alba*, *Pistacia vera*, *Punica granatum*, and *Vitis vinifera* are currently used in these workshops. Aiming to study the dye composition of wool samples dyed with the extracts of the selected biological sources, and the changes induced by the dyeing procedures in the original chemical composition of the plant extract, raw materials and dyed wool (by us and in the workshops) were analyzed by HPLC–DAD and UHPLC–HRMS/MS. In solutions extracted from the textiles, the main yellows for *E. laevigata* are luteolin-*O*-glycosides. In the other plant sources, the main chromophores are based on 3-*O*-glycosides of kaempferol, quercetin, and isorhamnetin. In pistachio hulls, myricitin derivatives were detected and we propose their use as markers. Generally, the solutions extracted from the wool displayed a higher amount of more polar compounds, but also a higher amount of aglycones. Importantly, the chromatographic profiles of the samples we prepared compared well with 17th c. yellows in Persian carpets, and therefore can be considered highly characterized references for the study of Persian yellows.

## 1. Introduction

### 1.1. Medieval Oriental Carpets: An Inherited Knowledge for Skilled Practitioners

In medieval times, dyeing was a craft exclusive to a few skilled individuals who made brilliant, fast colors using inherited recipes [1,2]. The knotted-pile oriental carpet, a surface composed of warp, weft, and knot, was an artistic object as well as a luxury good and, as such, also an object of status [2]. Medieval Persian carpets were made with brilliant, fast colors from a handful of natural dyestuffs. These natural dyes give very attractive non-uniform colors, which present differences in shade and intensity around a certain hue that create the illusion of movement—a vibrato effect [2]. Although we have evidence that the sources for red and blue were widely traded [1], recent research has shown that yellow dye plants may have been regional [3,4,5]. This was corroborated in our case study on natural yellow dyes used in Isfahan, a province in the central part of Iran (covering an area of 107,029 km^2^). Our research focused on the study of natural colorants used to dye yellow in Persia, in Isfahan, where, in the 21st century, dyeing with natural dyes is still practiced in workshops by skilled masters. Our main goal was to provide reference chromatographic profiles for dyes that were used to dye textiles in yellow, or combined with other dyes such as madder to create orange and brown hues. Thus, these profiles refer to solutions extracted from textiles. In this work, extracts from reference materials were compared with those obtained from wool threads dyed in Isfahan workshops, and with published data on the characterization of yellows in Persian carpets. In the next section, we describe how the plant sources were selected for this study and the main yellow dyes found in their extracts. This description is followed by a summary of the chromophores and plant sources that have been identified in literature as being used to dye yellow in historical artworks, in particular Persian carpets. Whenever possible, we selected data collected by HPLC-DAD-MS (high-performance liquid chromatography equipped with diode array and mass spectrometry detectors) using mild extraction procedures that preserved the integrity of the natural yellow chromophores, avoiding hydrolysis of the glycosidic linkages [3,5].

### 1.2. Yellow Flavonoids Extracted from Plants in Persia and 21st c. Iran

**Figure 1:**
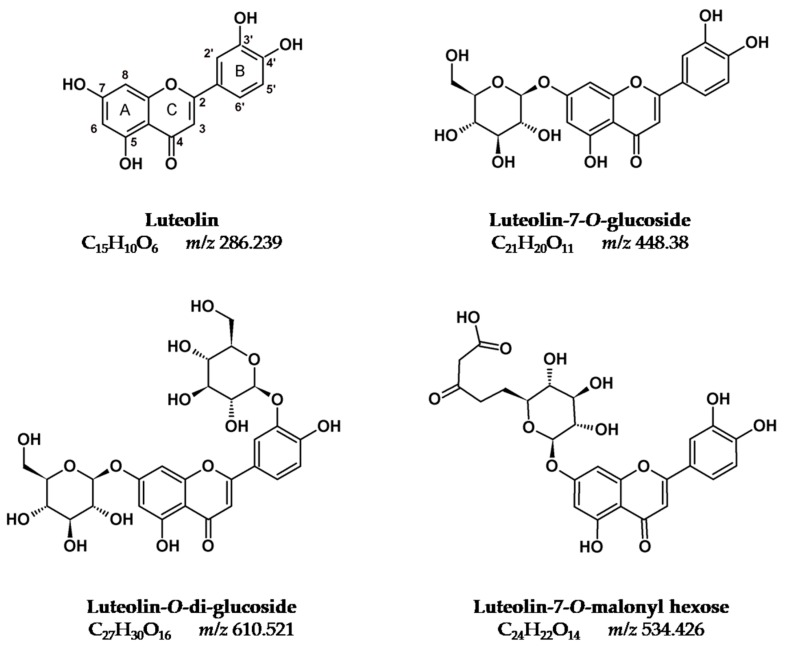
Luteolin-based chromophores (flavones).

**Figure 2:**
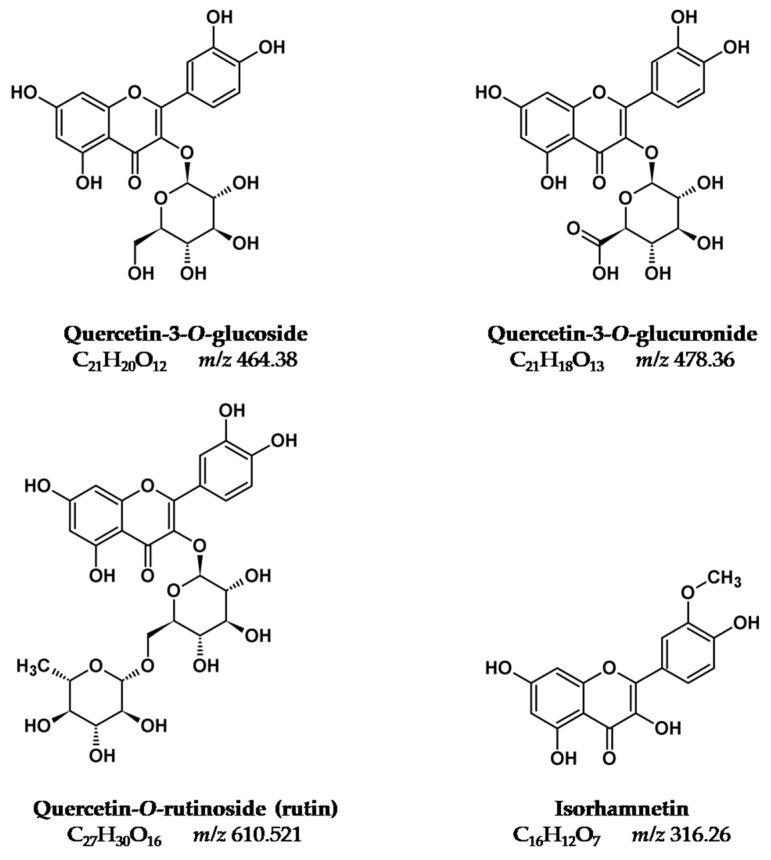
Quercetin-based chromophores (flavonols).

**Figure 3:**
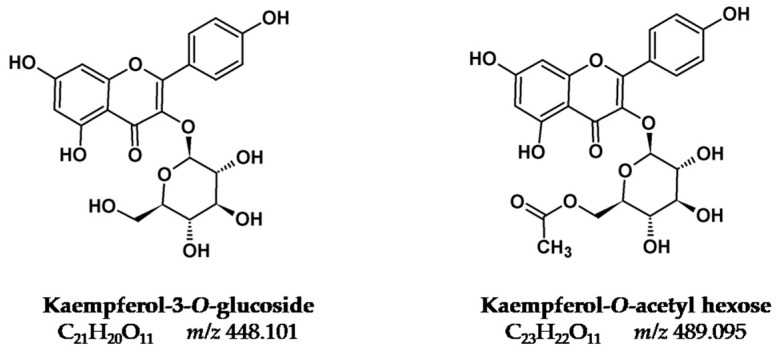
Kaempferol-based chromophores (flavonols).

Recently we prepared a review on plants that were used as sources of natural yellow dyes in Persian carpets [6], Figure 1, Figure 2 and Figure 3. The plants studied in the present work were selected based on that review (in particular, on data gathered regarding Persian and Iranian flora sources [7,8]), on recent characterization of yellows on Persian textiles, and on interviews with the few remaining dye masters in workshops located in Isfahan (central Iran) [9]. Cross-referencing the data gathered, we arrived at the list of nine plants that is found in Table 1. The species selected were in agreement with what is described in the reference book by Dominique Cardon [1], and with recent research carried out by Richard Laursen’s group at Boston University [3,4,10,11,12]. The fundamental yellow chromophores that were extracted from these plants are flavonoids based on the flavone and flavonol chromophore, Figure 1, Figure 2 and Figure 3. In literature, flavone-based yellows are considered more stable than flavonols, but the latter might be stabilized by transforming the OH group in position 3 into an *O*-glycoside [5,11].

Most of the plant extracts have been analyzed by HPLC-DAD or HPLC-DAD-MS [4,5,6,13,14,15,16], and, for some, accurate quantifications of the yellow flavonoids have been published [17,18]. On the other hand, chromatographic profiles of extracts from textiles dyed with these plants could only be found in cultural heritage studies for *D. semibarbatum,* Table 1. For this biological source, we had access to both the characterization of the plant extracts and of the solutions extracted from textiles [19]. For the other natural sources studied in this work, data on the yellow chromophores were only available for the plant extracts. Yellows present in the leaves of *Pistacia vera* were also analyzed by Laursen’s group, but they are not described here, because it is the pistachio hull that is used in Isfahan workshops [6]. In this group, Mouri et al. studied by HPLC-MS, *D. semibarbatum, P. ferulacea,* and *V. vinifera* extracts, which were obtained using water: methanol (1:1, *v*:*v*) [4], Table 1. *Delphinium semibarbatum* is characterized by nearly equimolar amounts of the 3-*O*-glucosides of kaempferol, quercetin, and isorhamnetin [4] (Figure 2 and Figure 3). Flavonol 3-*O*-glycosides were also the yellows found in all the twelve species of *Prangos* analyzed, displaying as major compounds 3-*O*-glucuronides of quercetin and isorhamnetin, or rutin [4] (Table 1). The first was also found to be the main yellow chromophore in extracts of *Vitis vinifera* leaves [4] (Table 1). Hmamouchi et al. [14] also studied *Vitis* leaves that were extracted with water: methanol (80:20, *v*:*v*); the aglicones were identified by HPLC chromatograms obtained at 340 nm by comparison with references, whereas sugars were analyzed by TLC; together with 3-*O*-gycosides of quercetin, the presence of apigenin-7-glucoside and luteolin-7-glucoside was also reported, which agreed with the analysis carried out by TLC by Böhmer et al. after hydrolysis with strong acids such as sulfuric or hydrochloric acids [15].

For pomegranate and *Eremostachys* species, the data available on yellow flavonoids is scarce. In the case of *Punica granatum*, this is because extractions were carried out in strong acidic media, and for this reason, the compounds identified were mainly flavonoid aglycones [15,16]. More recently, dihydrokaempferol-hexose was identified by HPLC-MS [17]. The extracts of its peel are dominated by the presence of polygalloyl esters of glucose (being punicalagin a marker for *Punica)*, and gallic and ellagic acids [17,18]. The main aglycones are listed in Table 1. For *Eremostachys* spp., luteolin-7-*O*-rutinoside was identified by Asnaashari et al. [20]; chromatograms were collected at 220 nm and the identification was performed via proton nuclear magnetic resonance (H-NMR; Table 1).

For mulberry leaves and pistachio hulls, a complete identification of the main yellow flavonoids together with accurate quantifications was published by Dugo et al. and Ersan et al., respectively [20,21]. For *Morus alba*, the flavonoid profile is dependent on the cultivars, as shown by Dugo et al.; for the morettiana cultivar, the two main yellow flavonoids were identified as rutin and isoquercitrin (quercetin 3-glucoside), in agreement with previous studies by Katsube et al. [21]. This author identified quercetin 3-(6-malonyl)-glucoside as the major flavonol glycoside, together with rutin and isoquercitrin, by HPLC-MS and H-NMR. For the korin cultivar, Dugo et al. observed a distribution over a wider number of flavonol glycosides, including kaempferol 3-*O*-glycosides, which were present in lower relative concentrations when compared to the morettiana cultivar [22] (Table 1).

Ersan et al. showed that flavonol glycosides comprise 5.7–16.3% of total phenolic constituents in pistachio hulls, anacardic acids being the major compounds (64.6–80.4% of total phenolics), followed by gallotannins (13.4–21.2%), such as β-glucogallin, gallic acid, and penta-*O*-galloyl-β-D-glucose [23] (Table 1). Quercetin 3-*O*-galactoside and quercetin 3-*O*-glucuronide were found to be the major yellow flavonoids together with quercetin 3-*O*-glucoside [23] (Table 1). As minor compounds, Ersan et al. tentatively identified myricetin 3-*O*-galactoside, myricetin hexuronide, myricetin hexosides, quercetin pentoside, quercetin hexosides, and traces of kaempferol hexosides and pentoside [23].

In summary, with the exception of *Eremostachys* species, characterized by a flavone chromophore of the luteolin type (Figure 1), in all the other plants, the main chromophores are based on flavonol 3-*O*-glycosides, which display a higher stability to light when compared to the parent aglycones shown in Figure 2 and Figure 3. For this reason, Mouri et al. concluded that “the dried plant can be used directly for dyeing and no precautions need be taken in drying it” (as is the case with *Sophora japonica*); this was possibly one of the reasons why these plants were selected in the past to dye textiles [4]. It is also interesting to note that in two of the six plants, in the parts chosen to be extracted (peel for pomegranate and hull for pistachio), the phenolic fraction is dominated by gallotannins (polygalloyl esters of glucose), and it is known that these compounds play an important auxiliary function in textile dyeing [1].

### 1.3. Yellow Dyes Analyzed in Persian Textiles

HPLC-DAD-MS remains one of the best methods available for identifying the colorants used in historical works, and can provide information as to where, when, and how historical and archaeological textiles were made, allowing their quantification when a calibration curve is used [24]. The main published works using HPLC-DAD or HPLC-DAD-MS for characterizing yellows in Persian textiles were carried out at Boston University, at the University NOVA of Lisbon (Department of Conservation and Restoration), and at the Metropolitan Museum of Art, MET, (New York) [19,25,26,27]. The main results are listed in Table 2 and show that in the Portuguese collections, the main chromophores are based on luteolin-7-O-glucosides, and in the MET collection, on flavonol 3-*O*-glycosides. In two of the publications, the authors proposed as plant sources *Reseda luteola* in [26], *Delphinium semibarbatum*, and *Carthamus tinctoria* in [19]. For more details, please see below.

One “small silk Kashan”, one “tree and animal”, and seven “Indo-Persian design” wool carpets from the 17th century, in the collection of “Museu Nacional de Arte Antiga”, were studied by Heitor et al. by HPLC-DAD and LC-MS [25]. Except for the small silk Kashan, for yellows, luteolin-7-*O*-glucoside was identified as the major chromophore, together with minor amounts of luteolin and apigenin. Orange colors were obtained by adding alizarin, in various amounts, to the previously described yellow. In the “small silk Kashan”, the yellow extracts were characterized by the presence of rutin, quercetin, and (iso)-rhamnetin-3-*O*-glucoside, as well as small percentages of luteolin and isoquercetin, suggesting golden rod or Persian berries as possible dye sources. In all the samples, aluminum ion was identified as the mordant by ICP-AES.

A “vine scroll” carpet held in the “Museu Nacional Machado de Castro” from the Safavid period (late 16th to early 17th century) composed of wool pile and silk wrap was analyzed by HPLC-DAD by Armindo et al. [26]. As in previous studies, luteolin-7-*O*-glucoside was detected as the main chromophore in yellows, together with minor amounts of luteolin, apigenin-7-*O*-glucoside, and apigenin (Figure 1 and Table 2). As in the previous case, alizarin was detected in orange colors admixed with the yellow dyes. ICP-AES analysis revealed aluminum ion as the mordant.

Santos et al. analyzed three Persian carpets which were knotted in wool on a silk foundation with metal (silver) thread decors [27]. These Safavid carpets, known as the “Salting carpets” were discovered in the palace of the Dukes of Bragança in Guimarães. The authors proposed *Reseda luteola* as the source for yellows and a combination of *Reseda luteola* and madder for orange (Table 2).

Persian velvets embellished with metal threads in the MET collection were studied by Shibayama et al. by HPLC-DAD [19]. These authors proposed the use of yellow larkspur *(Delphinium semibarbatum)* based on the identification of quercetin 3-*O*-hexoside, kaempferol 3-*O*-hexoside, and isorhamnetin 3-*O*-hexoside as the main flavonoids (Figure 1 and Table 2). In two samples, a combination of yellow larkspur with another plant source containing luteolin, luteolin 7-*O*-glucoside, and apigenin was found, a mixture similar to what was found in the “small silk Kashan” [25]. In another sample from a different velvet, kaempferol-3-*O*-glucoside was identified as the major chromophore together with minor amounts of carthamin and quinochalcone, which indicates that safflower plant (*Carthamus tinctoria*) may have been one of the dye sources.

In summary, in historical textiles, flavone chromophores such as luteolin-7-*O*-glucoside have been found as the main sources for yellows. Oranges were obtained by adding alizarin (probably extracted from madder, *Rubbia tinctorum*) to the yellows.

### 1.4. Design and Main Objectives

In this work, plant extracts were used to dye wool references with alum as mordant, based on the essential steps used in medieval times to dye textiles, and the extracts obtained from both the plant and from the dyed wool references were characterized by HPLC-DAD-HRMS/MS. The latter were compared with wool threads dyed in Isfahan workshops and with published data on the characterization of yellows in Persian carpets. Additionally, the chromatographic profiles obtained from the plant extracts were compared with extracts from the dyed textiles, and the changes observed are discussed. Chromatographic profiles for solutions extracted from wool textiles dyed with *E. laevigata*, *P. ferulacea*, *M. alba*, and *P. vera* (hull) were for the first time obtained and the changes induced by the dyeing procedure discussed. Extraction was performed using mild extraction procedures [4] to retain the glycoside fingerprint, avoiding decomposition in strong acidic media to their parent aglycons. This research will provide new knowledge on the past and current natural yellows used to dye in Iran, which is important information for their preservation for future generations.

## 2. Results and Discussion

### 2.1. Plants Selected and Collected in Iran

In the workshops in Isfahan, the sources for yellow were obtained from plants (Table 3). Based on the interviews in Isfahan dyeing workshops, three plants may be used in Iran as sources for yellow as a main color: *Delphinium semibarbatum*, *Eremostachys laevigata*, and *Prangos ferulacea;* however, in Isfahan workshops, only *D. semibarbatum* is used as the main source for saturated yellows (Table 3). The other biological sources are used to produce orange and brown colors or as co-dyes to create shades and/or intensify the yellow color (Table 3). Shades of yellow, orange, or brown colors are produced by combining *D. semibarbatum* with other dyes extracted from *Eremostachys laevigata, Morus alba, Vitis vinifera, Punica granatum*, and *Pistacia vera*. The color palette for yellows is thus variegated, as certain sources are used to create the main yellow colors and others are combined to produce oranges, dark yellows, and brownish colors.

**Figure 4:**
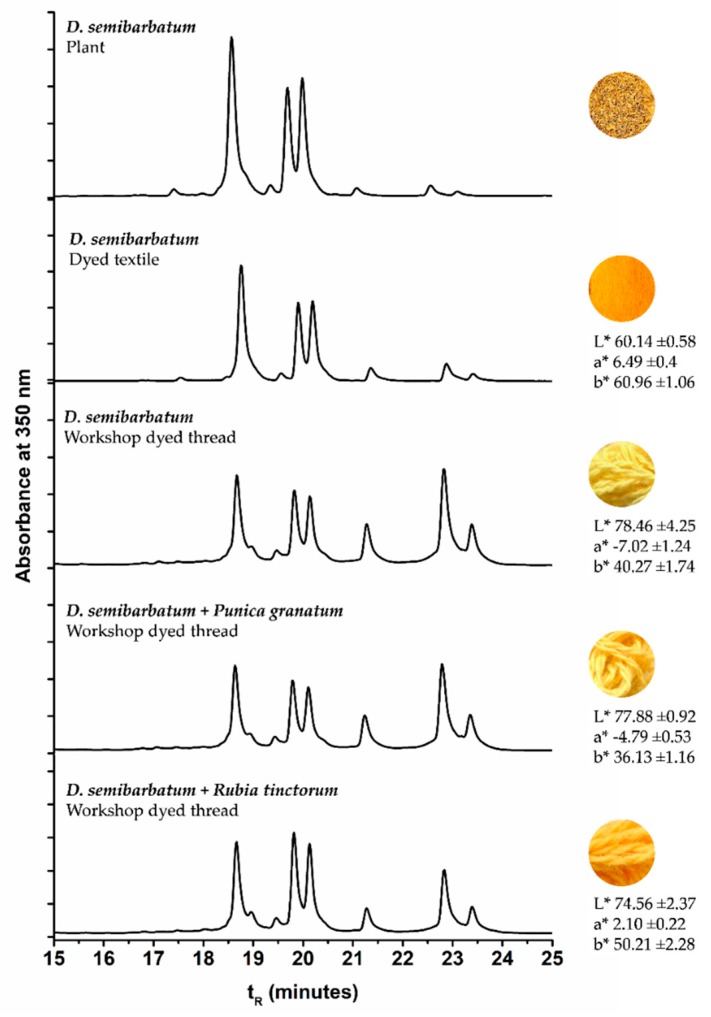
HPLC-DAD profiles for *D. semibarbatum* extract compared with the extracts from our reference sample and samples acquired at the workshop, together with the L*, a*, b* coordinates.

*Eremostachys laevigata* is the only species that produces luteolin-based chromophores (Figure 1); the crushed leaves and stems were supplied by R. Zakeri’s workshop, and it was used as such to produce the plant extracts to dye our wool samples (Figure 4). *Prangos ferulacea* came from the same workshop, as did crushed leaves and stems, and the flowers of *Delphinium semibarbatum* were obtained from a workshop located in Tudeshk. Both species are a source of quercetin and kaempferol glycosides (Figure 2 and Figure 3). The secondary colors*, Morus alba* leaves and *Pistacia vera* hulls, were collected from nature by the authors; *Punica granatum* was acquired in one of the workshops (Table 1 and Table 3).

### 2.2. Characterization by UHPLC- HRMS/MS and HPLC-DAD of the Main Chromophores in the Plants Collected in Isfahan and in Dyed Wool References

Extracts of plant material and dyed wool were analyzed by HPLC-DAD and UHPLC-HR tandem mass spectrometry to fully characterize the main yellow chromophores present in the biological sources. The information is summarized in Table S1. Compounds were identified based on their UV-VIS data and accurate *m/z* values released as deprotonated molecules [M–H]^−^, considering the accuracy and precision of the measurement parameters, such as error (ppm) and mSigma. Each molecular formula was validated by extracting the ion chromatograms from the raw data, and the accurate mass, isotopic, and fragmentation pattern were evaluated. The typical UV-VIS spectra obtained for major yellow components revealed a band I with a maximum of absorption between 350 and 370 nm, pointing to a flavonol structure, while a band II absorption around 268 nm indicated that the glycan parts of those chromophores were mostly *O*-glycosides (Table S2). When mentioned, compounds were confirmed by comparison with analytical standards or published data.

Delphinium semibarbatum: The main yellow chromophores present in this plant are O-glucosides of quercetin, kaempferol, and isorhamnetin, according to the study of Mouri et al. [4]. Two minor compounds with *m/z* 609.1461 and 447.0929 were also identified and attributed based on the MS/MS fragmentation patterns to a quercetin-O-di-glycoside and to a kaempferol-O-hexoside, respectively. The latter was assigned to a kaempferol structure based on the absence of the fragment *m/z* 133.0283 [^1,3^**B**]^−^, which is a diagnostic fragment of luteolin derivatives, in the ESI(-) tandem mass spectrum. The HRMS of plant extracts confirmed that D. semibarbatum contains a very low content of aglycones; however, in dyed wool extracts, the appearance of signals at *m/z* 301.0358, 285.0412, and 315.0519, indicates that during the preparation of the dyed wool references, deglycosylation of flavonol glycosides occurred.

*Eremostachys laevigata*: UV-VIS data of the flavonoid compounds present in this plant lay in the 340–350 nm region, suggesting the presence of flavone structures (Table S1-B). The HRMS/MS data confirmed that the main yellow components were luteolin-O-glycosides derivatives. Peaks at t_R_ 18.37 (6.18) and 20.28 (6.49) min were assigned to luteolin-pentoside-hexoside (*m/z* 579.1362) and luteolin-7-O-acetyl-glucoside (*m/z* 489,1040); the smaller one at t_R_ 19.38 (6.49) min was attributed to a luteolin-7-O-glucoside by comparision with a standard. Based on the fragmentation patterns observed in the MS/MS spectrum, peaks at 18.58 (6.37) and 21.55 (8.08) min were identified as luteolin-7-O-rutinoside and a manolyl derivative of luteolin glucoside, respectively. In the dyed wool extracts, the luteolin-manoyl-glucoside was found together with the luteolin aglycone.

*Morus alba*: The UV-VIS spectra obtained at 350 nm for plant and dyed wool extracts indicated that the main yellow compounds had a flavonol structure (Table S1-C). Based on the HRMS/MS spectra, the more intense signals at 18.63 (6.08) and 19.03 (6.41) min were identified as rutin and quercetin-3-O-glucoside, respectively, in accordance with previous results [21,22]. Minor peaks eluting at 19.77 (6.66), 19.87 (7.03), and 20.33 (7.43) min were assigned to O-glycoside derivatives of kaempferol. Although the more abundant yellow components were 3-O-glucoside flavonols, signals related to their aglycones were not found in the dyed wool extracts.

*Pistacia vera*: In this study, the plant extract was obtained from the pistachio hulls and not from leaves, as reported by Mouri et al. [4]. However, both parts of these specimens seemed to have similar flavonoid profiles, and the hull flavonoid profile assessed in our study was also in accordance with Ersan et al. [23]. HPLC profiles are shown in Table S1-D, having characteristic UV-VIS absorption around 350 nm pointing to flavonol glycosides. Peak 1 at t_R_ 17.25 (5.71) min was observed with the co-elution of two compounds with *m/z* 493.0628 and 479.0834 assigned to myricetin-O-glucuronide and quercetin-O-glucoside, respectively; peak **3** (R_t_ 6.45 min) co-elution of two compounds was also observed: quercetin-3-O-glucuronide (*m/z* 477.0675) and quercetin-3-O-glucoside (*m/z* 463.0883), the main constituents of the extract. The smaller peaks were attributable to quercetin and kaempferol glycoside derivatives. The MS/MS spectrum also exhibited a deprotonated molecule with *m/z* 447.0940 (t_R_ 6.98 min), which gave fragments with *m/z* 285.0406 and 284.0331, attibuted to a luteolin-7-O-glucoside by comparison with the standard. The peak at t_R_ 22.00 (9.16) min was assigned to luteolin aglycone. The identification of small amounts of luteolin and its glycoside derivative in pistachio hulls has been previously reported in the literature [23]. Since myricitin derivatives are not usualy found as yellow chromophores in plants, the two myricitin glycosides can be used as distinctive markers for plant identification.

*Prangos ferulacea*: As found in previous studies by the Laursen group [4], the primary yellow dyes present in the P. ferulacea extracts were glucuronic derivatives of quercetin and isorhamnetin, along with minor amounts of rutin. No signal of free aglycones was found in the HPLC chromatograms of the textiles (Table S1-E).

*Punica granatum*: HPLC extracts of the peel of pomegranate were dominated by the main peak eluting at t_R_ 19.38 (6.24) min assigned to ellagic acid, by comparison with the analytical standard. The three peaks eluting at lower retention times were identified as ellagitannins characteristic of this species: peak 1 was assigned as punicalin (MW 782), and the other two as forms alpha and beta of punicalagin (MW 1.084,74) which, in the ESI(−) tandem mass spectrum, appeared as double-charged deprotonated molecules at *m/z* 541.0272. The smaller peaks at higher retention times were due to ellagic acid derivatives. The HPLC profile displayed in Table S1-F indicates that during the preparation of dyed wool references, degradation of the ellagitannins occurred.

*Vitis vinifera*: The HPLC profile of grape leaf extract also presented a dominant peak at t_R_ 18.60 (6.46) min, identified as quercetin-O-glucuronide in accordance with reported literature data [4] (Table S1-G). The smaller peaks 2 and 3 were attributed as two kaempferol-3-O-glycoside isomers, isomer 3 being assigned to kaempferol-3-O-glucoside by comparison with the analytical standard. Although studies by the Laursen group reported that “in any event, no free aglycones were seen” [4], in our dyed wool extract, a signal at t_R_ 21.23 (9.27) min was clearly identified as quercetin, indicating that some deglycosylation of the quercetin–glucuronide can occur.

### 2.3. Comparison of the Chromatographic Profiles of the Main Chromophores in the Dyed Wool by HPLC-DAD

In Table 4, we compare the variation of the chromatographic profiles of plant and wool extracts for *E. laevigata*, *P. vera*, and *M. alba*, and in Table 5, we calculated the differences in the main peak ratios for all the species. As a general trend, we observed that the compounds that were more polar (more OH groups or more sugar substituents) were found in higher amounts in the wool extracts (Table 5). On the other hand, in the wool extracts, we also found the parent aglycones, which were not usually detected in the plant extracts (Table S1). It is possible that they were formed through the hydrolysis of the glycosidic bonds, by heating during the dyeing procedure.

### 2.4. Characterization of the Main Chromophores in Wool Threads of a Workshop in the Center of Iran

Samples were extracted and the solutions analyzed by HPLC-DAD-LRMS and HPLC-DAD (Figure 4). The colors of the threads were measured in the CIELAB color system (Figure 4). It was interesting to observe that in the workshop samples, the parent aglycones were present in very relevant amounts, reinforcing the trend already described in the extracts of our wool references. This means that, possibly, wool threads were dyed with higher temperatures and/or over longer periods then our reference samples. By HPLC-DAD-LRMS, it was possible to detect the presence of alizarin in the samples dyed with both *D. semibarbatum* and *R. tinctorum,* but in much lower amounts when compared with samples from 17th c. Persian carpets [24]. In these historical samples, alizarin was detected only in orange colors, where it may have applied in higher amounts than in the workshop samples. In the extracts of *D. semibarbatum* + *P. granatum,* it was possible to detect the presence of ellagic acid, but not of the punicalagin isomers. It is worth noting that when comparing the chromatographic profiles for *D. semibarbatum* + *R. tinctorum* and *D. semibarbatum* + *P. granatum,* the latter presented higher amounts of the parent aglycones (closer to the profile of samples dyed only with *D. semibarbatum*). As a general trend, all the extracts obtained from the workshop presented significantly higher concentrations of the parent aglycones when compared with our dyed wool extracts. On the other hand, our dyed samples, in terms of aglycone concentration, compared well with extracts obtained from 17th c. Persian carpets [25].

## 3. Materials and Methods

### 3.1. Materials

All solvents used were HPLC grade. Methanol was purchased from Merck, perchloric acid (HClO_4_) from ACS, and acetone ≥99.5% from Honeywell Riedel-de Haen. For all the chromatographic studies as well as dye extraction, Millipore ultrapure water was used. For UHPLC-HRMS, LC-MS–grade Optima methanol, acetonitrile, water, and LC-MS-grade formic acid were acquired from Fisher Scientific.

Quercetin (C_15_H_10_O_7_), luteolin (C_15_H_10_O_6_), kaempferol (C_15_H_10_O_6_), isorhamnetin (C_16_H_12_O_7_), apigenin (C_15_H_10_O_5_), ellagic acid (C_14_H_6_O_8_), luteolin-7-*O*-glucoside (C_21_H_20_O_11_), Luteolin-3′,7-di-O-glucoside (C_27_H_30_O_16_), kaempferol-3-*O*-glucoside (C_21_H_20_O_11_), quercetin-3-*O*-glucoside (C_21_H_19_O_12_), quercetin-3-*O*-glucuronide (C_21_H_18_O_13_), and rutin (C_27_H_30_O_16_) analytical standards were purchased from Extrasynthese.

### 3.2. The Plants: Collection and Preparation

**Figure 5:**
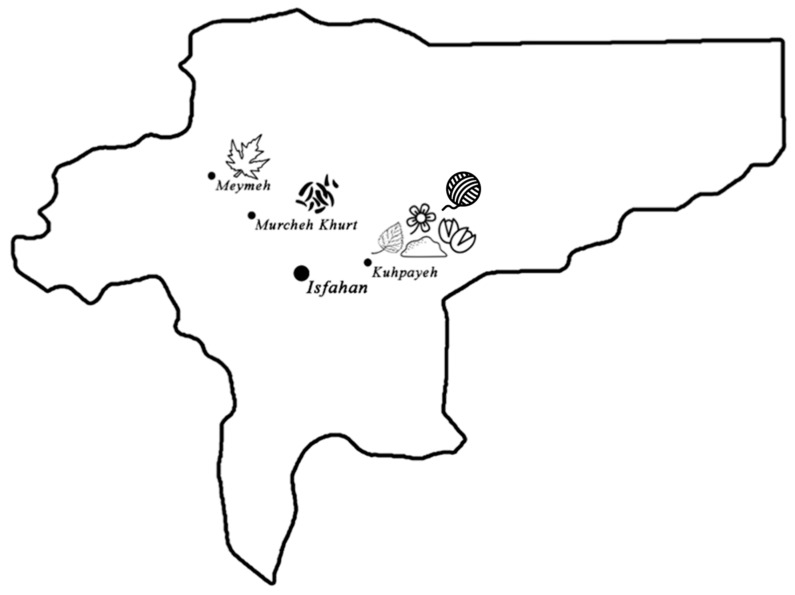
Geographical distribution of the collected plant sources used to dye yellow in Isfahan province, Iran [31].

Four plants were obtained from a workshop located in Isfahan on August 2016 (a central province in Iran): the flowers of *Delphinium semibarbatum* [28]*,* crushed leaves and stems of *Prangos ferulacea* and *Eremostachys laevigata*, and powdered peel of *Punica granatum*. The first two species were obtained in R. Zakeri’s workshop, Murcheh Khvort [29] (coordinates 33°05′24.7”N 51°28′40.8”E), while the latter two were acquired from Banitaba’s workshop (coordinates 32°41′37.9”N 52°43′31.2”E) of Tudeshk [30] (Figure 5).

The other three species were collected from nature in the province of Isfahan with different coordinates: the tree leaves of *Morus alba* and *Vitis vinifera* andthe hull of the fresh *Pistacia vera* were collected from geographical coordinates of 32°42′22.4”N 52°43′43.5”E, 33°26′48.6”N 51°10′14.7”E, and 32°51′37.1”N 53°05′11.5”E, respectively, Figure 5. These samples were dried spread in a tray, in the dark, in a ventilated area, at 30–40 °C.

### 3.3. Preparation of Dyed Wool References

In this work, wool references were mordanted with Al^3+^ and dyed with the plant extracts once only following a model procedure based on the steps necessary to dye yellow in medieval times that were adapted by Dominique Cardon [1]. According to the protocol provided by D. Cardon, 5 × 5 cm^2^ of unbleached woven wool fabric was pre-mordanted with 16% (mass of the wool) alum (KAl(SO_4_)_2_·12H_2_O) and 2% (mass of the wool) crude red tartar (KC_4_H_5_O_6_). The textiles were boiled for two hours. After being taken out of the mordant bath, the mordanted textile was allowed to cool down for about one day, and it was then washed in water.

Dry plant material (1 g) was placed in 100 mL of water and heated until the bath was nearly at boiling point (92–95 °C); the bath was then allowed to cool down to 25 °C with the plant material still in. The pre-mordanted textile was added at this point and the bath was heated again to boiling point (100 °C); it was allowed to boil containing both the plant material and the textile for 45 min.

### 3.4. Yellow Dyed Wools from Workshop in Center of Iran

Three yellow dyed wool yarn from H. Banitaba’s workshop (coordinates 32°41′37.9”N 52°43′31.2”E) near Isfahan were acquired. The samples ranged from light yellow to darker shades and were dyed using: (a) *Delphinium semibarbatum*; (b) *Delphinium* semibarbatum and *Punica granatum*; (c) *Delphinium* semibarbatum and *Rubia tinctorum*.

### 3.5. Extraction of Plants, Dyed Textiles or Fibers

In this work, we extracted two samples from the biological sources and two samples from the wool textiles and threads. The extractions were replicated twice for the HPLC-DAD analysis and once for the UHPLC-HRMS. Each replicate was analyzed at least twice by HPLC-DAD and UHPLC-HRMS/MS. The samples of plant specimens were extracted by placing 1 g of the dry plant material (as supplied by the workshop or as collected from nature) with 100 mL of methanol:water (70:30, *v*:*v*) and heating in a water bath at 60 °C for one hour, as described in Reference [4]. The extracts were filtered through cotton (a piece of cotton in a glass Pasteur pipette) and centrifuged at 12,000 rpm for about 10 min. The supernatant liquid was gently removed and centrifuged for about 5 min. Before analysis, the solution was diluted with methanol:water (70:30, *v*:*v*) if necessary.

The dye from the textiles was extracted by placing in a flask, 1 g of textile with a 3 mL solution of oxalic acid (0.2 M):methanol:acetone:water (0.1:3:3:4, *v*:*v*), as described by Reference [32]. The solution was left to evaporate and the residues were then dissolved in 400 μL of methanol/water, 7:3 (*v*/*v*); the tubes were centrifuged, and the upper 25 µL of the solution was removed for analysis.

### 3.6. HPLC-DAD and UHPLC-HRMS Equipment

The analysis of the extracts of both plant material and dyed wools was carried out in a Thermofinnigan Surveyor^®^ HPLC-DAD system with a Thermofinnigan Surveyor PDA (Thermofinnigan, San Jose, CA, USA), an autosampler, and a gradient pump. The sample separations were performed in a reversed-phase column, RP-18 Nucleosil column (Macherey-Nagel) with 5 µm particle size column (250 mm × 4.6 mm), with a flow rate of 1.7 mL/min with the column at a constant temperature of 35 °C. The samples were injected via a Rheodyne injector with a 25 μL loop. The elution gradient consisted of two solvents, A: methanol and B: 0.1% (*v*/*v*) perchloric acid aqueous solution. A gradient elution program was used of 0–2 min isocratic 7% A, 2–8 min linear gradient to 15% A, 8–25 min linear gradient to 75% A, 25–27 min linear gradient to 80% A, 27–29 min linear gradient to 100% A, and 29–30 min isocratic 100% A (10 min re-equilibration time). The eluted peaks were monitored at 350 nm.

Aliquots of 3 µL of both plant material and dyed wool extracts were also analyzed on a UHPLC Elute system coupled on-line with a quadrupole time-of-flight Impact II mass spectrometer equipped with an ESI source (Bruker Daltoniks, Bremen, Germany). Chromatographic separation was carried out on a RF-C18 Halo column (150 mm × 2.1 mm, 2.7 μm particle size, Advanced Material Technology). The mobile phase consisted of water (A) and acetonitrile (B), containing 0.1% formic acid, at a flow rate of 600 μL/min. The elution conditions were as follows: 0–18 min, linear gradient to 50% B; 18–20 min, linear gradient to 90% B; 20–23 min, isocratic 90% B; and 23–24 min, linear gradient to 0% B (followed by 11 min re-equilibration time). The column and the autosampler were maintained at 45 °C and 8 °C, respectively. High-resolution mass spectra were acquired in both ESI ionization modes. The mass spectrometric parameters were set as follows: end-plate offset: 500V; capillary voltage: 4.0 or −2.5 kV; nebulizer: 4 bars; dry gas: 8 L/min; heater temperature: 200 °C. Internal calibration was achieved with an ammonium formate solution introduced to the ion source via a 20 µL loop at the beginning of each analysis, using a six-port valve. Calibration was then performed using a high-precision calibration mode (HPC). Acquisition was performed in full scan mode in the *m*/*z* 100–1000 range and in a data-dependent MS/MS mode with an acquisition rate of 3 Hz using a dynamic method with a fixed cycle time of 3 s, and an *m*/*z* -dependent isolation window of 0.03 Da. Data acquisition and processing were performed using Data Analysis 4.2 software.

### 3.7. Colorimetry

To measure color, a portable Data Color International colorimeter spectrophotometer was used. Its measuring head’s optical system used diffuse illumination from a pulsed Xenon arc lamp over the 8mm diameter measuring area, with 0° viewing angle geometry. Color coordinates were calculated by defining the D65 illuminant and the 10° observer. The calibration was performed with a white bright standard plate and a total black standard. The color data were presented in the CIE-Lab system. The values represented are an average of three points.

## 4. Conclusions

Seven plants used for dyeing in yellow Persian textiles were studied by HPLC-DAD and UHPLC-HRMS/MS: *Delphinium semibarbatum*, *Eremostachys laevigata*, *Prangos ferulacea*, *Morus alba*, *Pistacia vera*, *Punica granatum*, and *Vitis vinifera*. The main yellows for *E. laevigata* were luteolin-*O*-glycosides derivatives (luteolin-pentoside-hexoside), this being the only plant in which this stable chromophore was identified. The other extracts were characterized by less stable 3-hydroxy flavone structures such as quercetin, kaempferol, and isorhamnetin, although always in the form of 3-*O*-glycosides, which will have a protective effect on the dye stability. For *Pistacia vera* (hulls), together with the main yellows (quercetin-3-*O*-glucoside and 3-*O*-glucuronide), myricitin derivatives were also detected as minor compounds, and we propose that they may be used as markers for plant identification.

Overall, we observed that the extracts from the wool samples displayed a higher amount of more polar chromophores, but, at the same time, in most of the extracts, a small amount of the parent aglycones was also detected (that were not present in the plant extracts). Mild extraction methods were used to prevent hydrolysis of the glycosidic linkages, so hydrolysis was possibly a result of the temperature used during the dyeing [3,33].

Beside our dyed wool references, we were also able to analyze samples from a workshop active in Isfahan: wool threads dyed with *Delphinium semibarbatum*, as a single dye or applied together with *Rubia tinctorum* or *Punica granatum*. Our analysis showed that the threads were in fact dyed with these plant sources. Interestingly, in the workshop samples, a high proportion of aglycones was detected. Moreover, when comparing our *Delphinium semibarbatum* extracts and the workshop extracts with previous studies on 17th c. Persian carpets, the profiles compared better with our dyeing procedure, i.e., parent aglycones were not present in high concentrations. This indicates that even when using similar natural sources for yellows, the methods used to dye in the workshops are different; possibly the dye baths are heated at higher temperatures or for longer time periods. It is also possible to conclude that our wool dyed samples may be used as highly characterized references in future research work.

As future work, it would be interesting to gather information on other workshops established in other regions of Iran, to verify whether the main biological sources for yellows are also flavonol-based or if other, flavone-based natural sources are preferred.

## Figures and Tables

**Table 1:** Main components described in literature for the plant sources. Plant origin, analytical, and extraction methods are described.

Plant Source	Reference	Origin	Equipment	Extraction	Main Yellow Flavonoids ^1^
*Delphinium semibarbatum*	Mouri et al. [4]	Uzbekistan, Turkey	HPLC-DAD-MS	MeOH: H_2_O (1:1, *v*:*v*) @65 °C	Kae-3-*O*-glu, Que-3-*O*-glu, Irh-3-*O*-glu
*Prangos* spp. (12 species)	Mouri et al. [4]	Iran	HPLC-DAD-MS	MeOH: H_2_O (1:1, *v*:*v*) @65 °C	Que-3-*O*-glr, Irh-3-*O* glr
*Vitis vinifera* (leaves)	Mouri et al. [4]	Iran	HPLC-DAD-MS	MeOH: H_2_O (1:1, *v*:*v*) @65 °C	Que-3-*O*-glr, other flavonol glycosides (minor amounts)
*Punica granatum* (peel)	El-Hadary and Fawzy Ramadan [16]	Egypt	HPLC-DAD	MeOH: H_2_O (4:1, *v*:*v*) @ rt	Que, Kae-3-(2-*p*-coumaroyl) glu, naringin, Api-6-rha- 8-galactose, Lut-7-glu
*E. azerbaijanica* (aerial parts)	Asnaashari et al. [20]	Iran	HPLC @220 nm; NMR	*n*-hexane, CH_2_Cl_2_ and MeOH	Lut-7-*O*-rut
*Morus alba* (leaves)	Katsube et al. [21]	Japan	HPLC-MS, NMR	EtOH:H_2_O	Que 3-(6-malonyl)-glu, rutin, Que-3-*O*-glu
*Morus alba* var. *korin*, *morettiana* (leaves)	Dugo et al. [22]	Italy	HPLC-DAD-MS	EtOH (95%) @ rt	korin: Kae-3-*O*-rha-glu, Kae-3-*O*-glu, morettiana: rutin, isoquercetin
*Pistacia vera* (hull)	Ersan et al. [23]	Turkey	HPLC-DAD-MS, UHPLC-DAD-ELSD	MeOH:H_2_O:HCOOH (80:19:1, *v*:*v*:*v*)	Que-3-*O*-galactoside, Que-3-*O*-glr, Que-3-*O*-glu, Que-galloyl hexoside Que-pentoside

^1^ Abbreviations: Que, quercetin; Kae, kaempferol; Lut, luteolin; Api, apigenin; Irh, isorhamnetin; glu, glucoside; glr, glucuronide; gly, glycoside.; hex: hexoside; pent: pentoside.

**Table 2:** Yellow flavonoids identified in Persian textiles, analytical methods used in their identification, and number of samples analyzed.

Dye Source	Reference	Location ^1^	Equipment	Artwork ^2^	Main Yellow Flavonoids
Luteolin based	Heitor [25]	MNAA	HPLC-DAD, LC-MS	16th c. wool carpets, 9 samples	Lut-7-*O*-glu low amounts of Lut, Api
*R. cartharicus* or *Solidago virgaurea*	Heitor [25]	MNAA	HPLC-DAD, LC-MS	16th c. silk carpet, 5 samples	Rutin, quercetrin, (iso)-rhamnetin-3-*O*-glu, and low percentage of Lut, isoquercitin
Luteolin based	Armindo et al. [26]	MNMC	HPLC-DAD	Late 16th c. wool carpet, 2 samples	Lut-7-*O*-glu, low amounts of Lut, Api-7-*O*-glu, Api
*Reseda luteola*	Santos [27]	Palace of Bragança	HPLC-DAD HPLC-MS	15–17th c. wool carpet, 7 samples	Lut-di-*O*-glu, Lut-7-*O*-glu, Api-7-*O*-glu, Lut
*Delphinium semibarbatum*	Shibayama et al. [19]	MET	HPLC-PDA	16–18th c. silk velvet, 13 samples	Que3-*O*-hexoside, Kae-3-*O*-hexoside, and Irh-3-*O*-hexoside
*D. semibarbatum* + *R. luteola*	Shibayama et al. [19]	MET	HPLC-PDA	16–18th c. silk velvet, 2 samples	Lut, Lut-7-*O*-glu, Api
Unknown yellow dye + *Carthamus tinctoria*	Shibayama et al. [19]	MET	HPLC-PDA	16–18th c. silk velvet, 1 sample	Kae-3-*O*-glu, minor amounts of quinochalcone and carthamin

^1^ Abbreviations: MNAA, Museu Nacional de Arte Antiga (Portugal); MNMC, Museu Nacional Machado de Castro (Portugal); Palace of Bragança, Palace of the Dukes of Bragança (Portugal); MET, Metropolitan Museum of Art (New York, USA). ^2^ Samples were extracted using “soft” extraction methods. For more details, please see references.

**Table 3:** Plant sources and parts used to dye yellow in Isfahan workshops, together with the place and date of their acquisition. Prangos may be used as main yellow in other regions of Iran.

Scientific Name	Icon ^§^	Common Name	Color Type	Parts of the Plant Used	Acquired/Collected
*Delphinium semibarbatum*	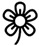	Yellow larkspur	Main	Flower	H. Banitaba’s workshop/ Tudeshk, August 2016
*Eremostachys laevigata*	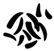	Desert rod	Main	leaf, stem (crushed)	R. Zakeri’s workshop /Murcheh khvort, August 2016
*Prangos* *ferulacea*	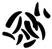	Prangos	Secondary	leaf, stem (crushed)	R. Zakeri’s workshop /Murcheh khvort, August 2016
*Punica granatum*	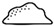	Pomegranate	Secondary	Peel (powder)	H. Banitaba’s workshop/ Tudeshk, August 2016
*Morus alba*	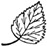	White mulberry	Secondary	leaf	Collected August 2016
*Pistacia vera*	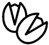	Pistachio	Secondary	Hull	Collected August 2016
*Vitis vinifera*	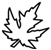	Vine	Secondary	leaf	Collected August 2016

^§^ Icons as used in Figure 4. The icons for *E. laevigata*, *P. ferulacea*, and *P. granatum* do not identify the plant, but that it was acquired as a crushed or powdered material.

**Table 4:** HPLC-DAD profiles for the extracts of the plant (upper) and dyed wool (lower) for *Eremostachys laevigata, Morus alba*, and *Pistacia vera.* The retention times, t*_R_*, for the main chromatographic peaks are given together with the wavelengths of the main absorption bands.

Name	Chromatogram @350 nm	*t*_R_ (min)	*λ*_max_ (nm)	Main Chromophores
***Eremostachys laevigata***	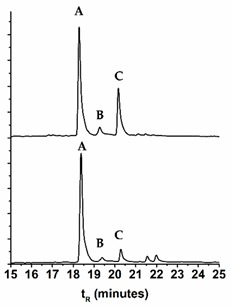			
		18.15	254, 348	Lut- pen-hex
		19.15	256, 345	Lut-7-*O*-glu
		20.07	254, 350	Lut-acetyl-hex
		
		
		18.37	254, 349	
		19.38	256, 344	
		20.28	254, 350	
		
		
***Morus alba***	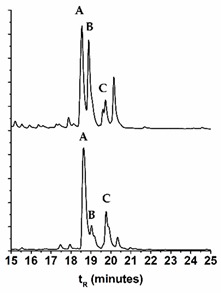			
		18.35	256, 356	Que-3-*O*-rutinoside
		18.70	255, 355	Que-3-*O*-glu
		19.43	264, 348	Lut-*O*-deoxyhex-hex
		
		
		18.63	256, 356	
		19.03	255, 355	
		19.77	264, 348	
		
***Pistacia vera***	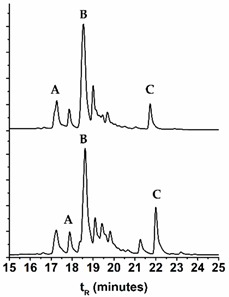				
		16.94	262, 357	Myr-3-*O*-glu	
		18.26	258, 355	Que-3-*O*-glr	
		21.58	254, 350	Lut	
			
			
		17.25	264, 358		
		18.62	258, 355		
		22.00	254, 350		
			

**Table 5:** Comparison of the main chromophores ^1^ extracted from the plants and the dyed wool: the ratios were obtained by normalizing the areas of the main chromophores, A B C, by the main peak in the chromatogram (HPLC-DAD). For more details, please see Table S2.

Name	Plant extract	Wool extract	Peak A	Peak B	Peak C
*D. semibarbatum*	A:B = 1.85 A:C = 1.49	A:B = 1.94 A:C = 1.55	Quer-3-*O*-Glc	Kae-3-*O*-Glc	Irh-*O*-Glc
*E. laevigata*	A:B = 5.92 A:C =2.04	A:B = 12.4 A:C = 6.24	Lut-pen-Hexose	Lut-7-*O*-Glc	Lut-acetyl-Hexose
*P. ferulacea*	B:A = 1.84	B:A = 2.01	Quer-3-*O*-Glr	Irh-3-*O*-Glr	-
*M. alba*	A:B = 1.08 A:C =1.83	A:B = 3.67 A:C = 4.18	Quer-3-*O*-rutinoside	Quer-3-*O*-Glc	Kae-*O*-acetyl-Hexose
*P. vera*	B:A = 6.26 B:C = 4.80	B:A = 3.83 B:C = 2.84	Myr-3-*O*-Glc	Quer-3-*O*-Glr	Lut
*P. granatum*	C:A = 1.48 C:B’ = 0.89	C:A = 1.84 C:B = 2.04	Punicalin	Punicalagin B	Ellagic acid
*V. vinifera*	A:B = 6.62	A:B = 7.17	Quer-*O*-Glr	Kae-3-*O*-Glc	-

*^1^* Glc—glucose and Glr—Glucuronide.

## Supplementary Materials

The following are available online, Figure S1: Morphology of *Pistacia vera*; Table S1: HPLC-DAD and LC-ESI(−)-HRMS/MS characterization of the main yellow chromophores present in plant (Pl) and dyed wool (Tx) extracts. Table S2. Chromatograms of both plant and dyed wool extracts, retention times and correspondent absorbance maxima (λ_max_) and mass, as well as UV-Vis spectra of the main peaks (A, B and C). Table S3. Flavonoid references: common and scientific names, molecular structure, retention time, absorbance maxima and UV-VIS spectra as acquired by HPLC-DAD.
