# Supplementary material for: Traditional Yellow Dyes Used in the 21st Century in Central Iran: The Knowledge of Master Dyers Revealed by HPLC-DAD and UHPLC-HRMS/MS
Source: Molecules. 2020 Feb 18;25(4):908. doi: 10.3390/molecules25040908 (PMC7070888; doi:10.3390/molecules25040908)
Supplement: Supplementary file 1 [file molecules-25-00908-s001.zip › Supplemental material/Supplementary table S1.pdf]

Table S1. HPLC-DAD and LC-ESI(-)-HRMS/MS characterization of the main yellow chromophores present in plant (Pl) and dyed wool (Tx) extracts.

	**Textile t_R (min)**		**λmax (nm)**	**MF**	**$[M-H]^-$**		**Δ ppm**	**MS/MS [*m/z* (Δ ppm)(attribution)]**	**Proposed compound**	**Pl**	**Tx**
	**HPLC**	**UHPLC**			**Meas. *m/z***	**Cal. *m/z***					
Delphinium semibarbatum	17.55	5.85	256, 356	$C_{27}H_{30}O_{16}$	609.1467	609.1461	-1.0	300.0277 (-0.4) $[Y_0-H^{\bullet}]^{\bullet -}$ $[C_{15}H_8O_7]^{\bullet -}$	Que-3-*O*-dexoxy-hex	1	1
	**18.75**	**6.42**	**255, 355**	$C_{21}H_{20}O_{12}$	**463.0876**	**463.0882**	**1.2**	**301.0347 (2.1) $[Y_0]^-$ $[C_{15}H_9O_7]^-$ 300.0277 (-0.4) $[Y_0-H^{\bullet}]^{\bullet -}$ $[C_{15}H_8O_7]^{\bullet -}$ 271.0250 (-0.7)$[Y_0-2H-CO]^-$ $[C_{14}H_7O_5]^-$**	**Que-3-*O*-glu***	**2**	**2**
	19.55	6.79	264, 348	$C_{21}H_{20}O_{11}$	447.0929	447.0933	0.8	284.0328 (-0.5) $[Y_0-H^{\bullet}]^{\bullet -}$ $[C_{15}H_8O_6]^{\bullet -}$ 255.0303 (-1.3)$[Y_0\text{-}2H-CO]^-$ $[C_{14}H_7O_5]^-$	Kae-hex	3	3
	**19.90**	**7.04**	**264, 347**	$C_{21}H_{20}O_{11}$	**447.0931**	447.0933	**0.5**	**285.0402 (0.8) $[Y_0]^-$ $[C_{15}H_9O_6]^-$ 284.0328 (-0.5) $[Y_0-H^{\bullet}]^{\bullet -}$ $[C_{15}H_8O_6]^{\bullet -}$ 255.0291 (3.3) $[Y_0\text{-}2H-CO]^-$ $[C_{14}H_7O_5]^-$**	**Kae-3-*O*-glu***	**4**	**4**
	**20.19**	**7.11**	**254, 354**	$C_{22}H_{22}O_{12}$	**477.1040**	**477.1038**	**-0.3**	**315.0505 (1.6) $[Y_0]^-$ $[C_{16}H_{11}O_7]^-$ 314.0433 (-0.2) $[Y_0-H^{\bullet}]^{\bullet -}$ $[C_{16}H_{10}O_7]^{\bullet -}$ 285.0409 (-1.6)$[Y_0\text{-}2H-CO]^-$ $[C_{15}H_9O_6]^-$**	**Irh-3-*O*-glu**	**5**	**5**
	21.35	9.27	254, 371	$C_{15}H_{10}O_7$	301.0359	301.0354	-1.7	178.9980 (-0.4) $[^{1,2}A]^-$ $[C_8H_3O_5]^-$ 151.0037 (-0.7) $[^{1,2}A-CO]^-$ $[C_7H_3O_4]^-$ 121.0280 (10.0) $[^{1,2}B]^-$ $[C_7H_5O_2]^-$	Que*	n.a.	6
	22.87	10.63	264, 366	$C_{15}H_{10}O_6$	285.0413	285.0405	-2.9	151.0036 (0.7) $[^{1,3}A]^-$ $[C_7H_3O_4]^-$	Kae*	n.a.	7
	23.40	10.91	254, 370	$C_{16}H_{12}O_7$	315.0519	315.0510	-2.7	300.0282 (-2.0) $[C_{15}H_8O_7]^-$ 151.0037 (-0.7) $[^{1,2}A-CO]^-$ $[C_7H_3O_4]^-$	Irh*	n.a.	8

	Textile t_R (min)		λmax (nm)	MF	[M-H]$^-$		Δ ppm	MS/MS	Proposed compound	Pl	Tx
	HPLC	UHPLC			Meas. *m/z*	Cal. *m/z*		[*m/z* (Δ ppm)(attribution)]			
Eremostachys laevigata	**18.37**	**6.18**	**254, 349**	**$C_{26}H_{28}O_{15}$**	**579.1362**	**579.1355**	**-0.7**	**285.0404 (-0.2) $[Y_0]^-$ $[C_{15}H_9O_6]^-$**	**7-O-[β-D-arabinopyranosyl-(1→6)-β-D-glucosyl]luteolin**	**1**	**1**
	-	6.37	n.a.	$C_{27}H_{30}O_{15}$	593.1519	593.1512	-0.7	447.0936 (-0.3) $[C_{21}H_{19}O_{11}]^-$<br>285.0404 (-1.3) $[Y_0]^-$ $[C_{15}H_9O_6]^-$<br>284.0328 (-0.5) $[Y_0\text{-}H^{\bullet}]^{\bullet-}$ $[C_{15}H_8O_6]^{\bullet-}$	Luteolin-7-O-rutinoside	n.a.	n.a.
	**19.38**	**6.49**	**256, 345**	**$C_{21}H_{20}O_{11}$**	**447.0938**	**447.0933**	**-0.5**	**285.0406 (-0.3) $[Y_0]^-$ $[C_{15}H_9O_6]^-$<br>284.0331 (-1.6) $[Y_0\text{-}H^{\bullet}]^{\bullet-}$ $[C_{15}H_8O_6]^{\bullet-}$**	**Lut-7-O-glu***	**2**	**2**
	**20.28**	**7.18**	**254, 350**	**$C_{23}H_{22}O_{12}$**	**489.1040**	**489.1038**	**-0.1**	**447.0932 (0.1) $[C_{21}H_{19}O_{11}]^-$<br>285.0407 (-0.8) $[Y_0]^-$ $[C_{15}H_9O_6]^-$<br>284.0328 (-0.7) $[Y_0\text{-}H^{\bullet}]^{\bullet-}$ $[C_{15}H_8O_6]^{\bullet-}$**	**Lut-acetyl-hex**	**3**	**3**
	21.55	8.08	254, 350	$C_{24}H_{22}O_{14}$	533.0939	533.0937	-0.3	489.10438 (0.1) $[C_{23}H_{22}O_{12}]^-$<br>285.0408 (-1.0) $[Y_0]^-$ $[C_{15}H_9O_6]^-$	Lut-manoyl-hex	n.a.	4
	21.98	9.15	254, 350	$C_{15}H_{10}O_6$	285.0408	285.0405	-0.4	151.0034 (-0.7) $[^{1,3}A]^-$ $[C_7H_3O_4]^-$<br>133.0283 (9.0) $[^{1,3}B]^-$ $[C_8H_5O_2]^-$	Lut*	n.a.	5
Morus Alba	**18.63**	**6.08**	**256, 356**	**$C_{27}H_{30}O_{16}$**	**609.14670**	**609.1461**	**-1.5**	**301.0346 (2.6) $[Y_0]^-$ $[C_{15}H_9O_7]^-$<br>300.0278 (-0.4) $[Y_0\text{-}H^{\bullet}]^{\bullet-}$ $[C_{15}H_8O_7]^{\bullet-}$<br>271.0250(-0.5) $[Y_0\text{-}2H\text{-}CO]^-$ $[C_{14}H_7O_6]^-$**	**Que-3-*O*-rutinoside***	**1**	**1**

	Textile t_R (min)		λmax (nm)	MF	$[M\text{-}H]^-$		Δ ppm	MS/MS	Proposed compound	Pl	Tx
	HPLC	UHPLC			Meas. *m/z*	Cal. *m/z*		[*m/z* (Δ ppm)(attribution)]			
	**19.03**	**6.41**	**255, 355**	**$C_{21}H_{20}O_{12}$**	**463.0883**	**463.0882**	**-0.3**	**300.0276 (-0.1) $[Y_0\text{-}H^\bullet]^{\bullet-}$ $[C_{15}H_8O_7]^{\bullet-}$**<br>**271.0250 (-1.9) $[Y_0\text{-}2H\text{-}CO][C_{14}H_7O_5]^-$**	**Que-3-*O*-glu***	**2**	**2**
	**19.77**	**6.66**	**264, 348**	**$C_{27}H_{30}O_{15}$**	**593.1513**	**593.1512**	**-0.2**	**447.0935 (-0.6) $[C_{21}H_{19}O_{11}]^-$**<br>**285.0403 (-0.5) $[Y_0]^-$ $[C_{15}H_9O_6]^-$**<br>**284.0329(-0.8) $[Y_0\text{-}H^\bullet]^{\bullet-}$ $[C_{15}H_8O_6]^{\bullet-}$**<br>**255.0302(-1.1) $[Y_0\text{-}2H\text{-}CO]^-$ $[C_{14}H_7O_5]^-$**	**Kae-3-*O*-rutinoside**	**3**	**3**
	19.87	7.03	264, 347	$C_{21}H_{20}O_{11}$	447.0935	447.0933	-0.4	285.0397 (0.8) $[Y_0]^-$ $[C_{15}H_9O_6]^-$<br>284.0331 (-1.6) $[Y_0\text{-}H^\bullet]^{\bullet-}$ $[C_{15}H_8O_6]^{\bullet-}$<br>255.0303(-1.6) $[Y_0\text{-}2H\text{-}CO]^-$ $[C_{14}H_7O_5]^-$	Kae-3-*O*-glu*	4	4
	20.33	7.49	264, 348	$C_{23}H_{22}O_{12}$	489.1044	489.1º38	-1.1	285.0397 (2.7) $[Y_0]^-$ $[C_{15}H_9O_6]^-$<br>284.0329 (-0.9) $[Y_0\text{-}H^\bullet]^{\bullet-}$ $[C_{15}H_8O_6]^{\bullet-}$<br>255.0305(-2.6) $[Y_0\text{-}2H\text{-}CO]^-$ $[C_{14}H_7O_5]^-$	Kae-O-acetyl-hex	5	5
Pistacia vera	**17.25**	**5.71**	**264, 358**	**$C_{21}H_{18}O_{14}$**	**493.0628**	**493.0624**	**-0.8**	**317.0307 (-1.3)$[Y_0]^-$ $[C_{15}H_9O_8]^-$**<br>**299.0204 (-2.1) $[C_{15}H_7O_7]^-$**	**Myr-3-*O*-glr**	**1**	**1**
				**$C_{21}H_{20}O_{13}$**	**479.0834**	**479.0831**	**-0.6**	**316.0227(-0.6) $[Y_0\text{-}H^\bullet]^{\bullet-}$ $[C_{15}H_8O_8]^{\bullet-}$**<br>**287.0206(-3.0) $[Y_0\text{-}2H\text{-}CO]^-$ $[C_{14}H_7O_7]^-$**	**Myr-3-*O*-glu**		

Textile t_R (min)		λmax (nm)	MF	[M-H]$^-$		Δ ppm	MS/MS [*m/z* (Δ ppm)(attribution)]	Proposed compound	Pl	Tx
HPLC	UHPLC			Meas. *m/z*	Cal. *m/z*					
17.88	5.91	278, 354	$C_{28}H_{24}O_{16}$	615.0992	615.0992	-0.1	463.0887 (-1.0) $[Y_1]^-$ $[C_{21}H_{19}O_{12}]^-$<br>301.0349 (1.7) $[Y_0]^-$ $[C_{15}H_9O_7]^-$<br>300.0281 (-1.7) $[Y_0\text{-}H^\bullet]^{\bullet-}$ $[C_{15}H_8O_7]^{\bullet-}$<br>271.0260(-1.8)$[Y_0\text{-}2H\text{-}CO]^-$ $[C_{14}H_7O_6]^-$	Que-3-galloyl-glu	2	2
**18.62**	**6.45**	**258, 355**	$\mathbf{C_{21}H_{18}O_{13}}$	**477.0675**	**477.0675**	**-0.1**	**301.0356 (-0.7)** $[Y_0]^-$ $[C_{15}H_9O_7]^-$<br>**273. 0407 (-0.9))**$[Y_0\text{-}CO]^-$ $[C_{14}H_9O_6]^-$	**Que-3-O-glr**	**3**	**3**
			$C_{21}H_{20}O_{12}$	**463.0883**	**463.0882**	**-0.3**	**300.0220 (-4.8)** $[Y_0\text{-}H^\bullet]^{\bullet-}$ $[C_{15}H_8O_7]^{\bullet-}$<br>**271.0251(-1.2)** $[Y_0\text{-}2H\text{-}CO]^-$ $[C_{14}H_7O_5]^-$	**Que-3-O-glu**		
19.10	6.82	265; 358	$C_{20}H_{18}O_{11}$	433.0777	433.0776	-0.2	300.0285 (-4.4) $[Y_0\text{-}H^\bullet]^{\bullet-}$ $[C_{15}H_8O_7]^{\bullet-}$<br>271.0265(-4.8) $[Y_0\text{-}2H\text{-}CO]^-$ $[C_{14}H_7O_6]^-$	Que-3-*O*-pen	4	4
-	6.98	n.a.	$C_{21}H_{20}O_{11}$	447.0940	447.0933	-1.6	285.0406 (-0.3) $[Y_0]^-$ $[C_{15}H_9O_6]^-$<br>284.0331 (-1.6) $[Y_0\text{-}H^\bullet]^{\bullet-}$ $[C_{15}H_8O_6]^{\bullet-}$	Lut-7-O-glu*	n.a.	n.a.
19.82	7.69	266, 352	$C_{28}H_{22}O_{17}$	629.07837	629.0784	-0.4	477.0674 (0.1) $[Y_1]^-$ $[C_{21}H_{17}O_{13}]^-$<br>301.0357 (-1.1) $[Y_0]^-$ $[C_{15}H_9O_7]^-$	Que-galloyl-glr	5	5
**22.00**	**9.16**	**254, 350**	$\mathbf{C_{15}H_{10}O_6}$	**285.0411**	**285.0405**	**-2.4**	**151.0035 (-1.0)** $[^{1,3}A]^-$ $[C_7H_3O_4]^-$<br>**133.0284 (8.7)** $[^{1,3}B]^-$ $[C_8H_5O_2]^-$	**Lut***	**6**	**6**

	Textile t_R (min)		**λmax (nm)**	**MF**	**[M-H]$^-$**		**Δ ppm**	**MS/MS [*m/z* (Δ ppm)(attribution)]**	**Proposed compound**	**Pl**	**Tx**
	**HPLC**	**UHPLC**			**Meas. *m/z***	**Cal. *m/z***					
Prangos ferulacea	**18.60**	**6.43**	**256, 354**	$C_{21}H_{18}O_{13}$	**477.0681**	**477.0675**	**-1.3**	**301.0358 (-1.8) [Y_0]$^-$ [$C_{15}H_9O_7$]$^-$**	**Que-3-*O*-glr***	**1**	**1**
	**20.33**	**7.24**	**254, 352**	$C_{22}H_{20}O_{13}$	**491.0832**	**491.0831**	**-0.1**	**315.0515 (-1.4) [Y_0]$^-$ [$C_{16}H_{11}O_7$]$^-$ 271.0252 (-1.4) [Y_0-2H-CO]$^-$ [$C_{14}H_7O_6$]$^-$**	**Irh-3-*O*-glr**	**2**	**2**
Punica granatum	**3.73**	**2.24**	**257, 376**	$C_{34}H_{22}O_{22}$	**781.0521**	**781.0530**	**0.9**	**600.9899 (-0.5) [$C_{28}H_7O_{16}$]$^-$ 300.9993 (-1.1)[Y_0]$^-$ [$C_{14}H_5O_8$]$^-$**	**Punicalin**	**1**	**1**
	6.05	3.02	257, 377	$C_{48}H_{28}O_{30}$	541.0266#	541.0260	-0.6	[782.0564 (4.2) + 300.9995 (-1.6) [$C_{34}H_{22}O_{22}$]$^-$ + [$C_{14}H_5O_8$]$^-$	Punicalagin A	2	2
	**8.89**	**3.69**	**257, 378**	$C_{48}H_{28}O_{30}$	**541.0268 #**	**541.0260**	**-0.7**	**[782.0564 (4.2) + 300.9995 (-1.6) [$C_{34}H_{22}O_{22}$]$^-$ + [$C_{14}H_5O_8$]$^-$**	**Punicalagin B**	**3**	**3**
	15.51	5.46	256, 362	$C_{27}H_{22}O_{18}$	633.0629	633.0733	-0.8	463.0523 (-1.1) [$C_{20}H_{15}O_{13}$]$^-$ 300.9989 (0.4) [Y_0]$^-$ [$C_{14}H_5O_8$]$^-$	Galloy-HHDP-hex	4	4
	16.45	5.70	254, 377	$C_{19}H_{14}O_{12}$	433.0403	433.0412	0.9	300.9987 (1.0) [Y_0]$^-$ [$C_{14}H_5O_8$]$^-$ 299.9911 (0.2) [Y_0-H$^\bullet$]$^{\bullet-}$ [$C_{14}H_4O_8$]$^{\bullet-}$	Ellagic acid-xylopyranoside	5	5
	17.01	5.88	252, 359	$C_{20}H_{16}O_{12}$	447.0565	447.0569	0.4	300.9978 (4.1) [Y_0]$^-$ [$C_{14}H_5O_8$]$^-$ 299.9913 (-0.4) [Y_0-H$^\bullet$]$^{\bullet-}$ [$C_{14}H_4O_8$]$^{\bullet-}$	Ellagic acid-rhamnoside	6	6
	**19.38**	**6.24**	**250, 368**	$C_{14}H_6O_8$	**300.9996**	**300.9990**	**-0.6**	**273.0047 (-0.7) [$C_{13}H_5O_7$]- 257.0096 (-1.8) [$C_{13}H_5O_6$]- 229.0145 (-1.0) [$C_{12}H_5O_5$]-**	**Ellagic acid***	**7**	**7**

	Textile t_R (min)		λmax (nm)	MF	$[M-H]^-$		Δ ppm	MS/MS [*m/z* (Δ ppm)(attribution)]	Proposed compound	Pl	Tx
	HPLC	UHPLC			Meas. *m/z*	Cal. *m/z*					
Vitis vinifera	**18.60**	**6.46**	**256, 354**	$C_{21}H_{18}O_{13}$	**477.0675**	**477.0675**	**-0.1**	**301.0356 (0.6) $[Y_0]^-$ $[C_{15}H_9O_7]^-$**	**Que-*O*-glr***	**1**	**1**
	-	6.81	n.a.	$C_{21}H_{20}O_{11}$	447.0931	447.0933	0.3	284.0331 (-1.6) $[Y_0\text{-}H^\bullet]^{\bullet -}$ $[C_{15}H_8O_6]^{\bullet -}$<br>255.0300(-0.2)$[Y_0\text{-}2H\text{-}CO]^-$ $[C_{14}H_7O_5]^-$	Kae-glu isomer	n.a.	n.a.
	**19.80**	**7.02**	**264, 348**	$C_{21}H_{20}O_{11}$	**447.0933**	**447.0933**	**0.0**	**285.0404 (0.1) $[Y_0]^-$ $[C_{15}H_9O_6]^-$<br>284.0331 (-1.6) $[Y_0\text{-}H^\bullet]^{\bullet -}$ $[C_{15}H_8O_6]^{\bullet -}$<br>255.0306(-2.6) $[Y_0\text{-}2H\text{-}CO]^-$ $[C_{14}H_7O_5]^-$**	**Kae-3-*O*-glu***	**2**	**2**
	**21.23**	**9.27**	**254, 370**	$C_{15}H_{10}O_7$	**301.0357**	**301.0354**	**-1.0**	**151.0030 (4.4) $[^{1,3}A]^-$ $[C_7H_3O_4]^-$<br>121.0280 (10.0) $[^{1,2}B]^-$ $[C_7H_5O_2]^-$**	**Que***	**n.a.**	**3**

Abbreviations: Que, quercetin; Kae, kaempferol; Lut, luteolin; Irh, isorhamnetin; rha, rhamose; glc, glucoside; glr, glucuronide; gly, glycoside; deoxyhex, deoxyhexoside; hex, hexoside, pen, pentoside; rut, rutinoside.

Double charged deprotonated molecules $[M-2H]^{2-}$

* Identified with an analytical standard
