# Supplementary material for: Traditional Yellow Dyes Used in the 21st Century in Central Iran: The Knowledge of Master Dyers Revealed by HPLC-DAD and UHPLC-HRMS/MS
Source: Molecules. 2020 Feb 18;25(4):908. doi: 10.3390/molecules25040908 (PMC7070888; doi:10.3390/molecules25040908)
Supplement: Supplementary file 1 [file molecules-25-00908-s001.zip › Supplemental material/Supplementary table S2.pdf]

Plant				Textile			(Da)	
Delphinium semibarbatum								
	1	17.25	256, 355		17.55	256, 356	610	
	**2**	**18.43**	**255, 355**		**18.75**	**255, 355**	**464**	
	3	19.23	263, 347		19.55	264, 348	448	
	**4**	**19.58**	**264, 347**		**19.90**	**264, 347**	**448**	
	**5**	**19.89**	**254, 354**		**20.19**	**254, 354**	**478**	
	6	-	-		21.35	254, 371	302	
	7	-	-		22.87	264, 366	286	
	8	-	-		23.40	254, 370	316	
Eremostachys laevigata								
	**1**	**18.15**	**254, 348**		**18.37**	**254, 349**	**580**	
	**2**	**19.15**	**256, 345**		**19.38**	**256, 344**	**448**	
	**3**	**20.07**	**254, 350**		**20.28**	**254, 350**	**490**	
	4	-	-		21.55	254 350	534	
	5	-	-		21.98	254,350	286	
Morus alba								
	**1**	**18.35**	**256, 356**		**18.63**	**256, 356**	**610**	
	**2**	**18.70**	**255, 355**		**19.03**	**255, 355**	**464**	
	**3**	**19.43**	**264, 348**		**19.77**	**264, 348**	**594**	
	4	19.54	264, 347		19.87	264, 347	448	
	5	20.00	264, 348		20.33	264, 348	490	
Pistacia vera								
	**1**	**16.94**	**262, 357**		**17.25**	**264,358**	**480**	
	2	17.55	276, 353		17.88	278,354	616	
	**3**	**18.26**	**258, 355**		**18.62**	**258,355**	**478**	
	4	18.74	265, 358		19.10	265, 358	434	
	5	19.45	267, 350		19.82	266, 352	630	
	**6**	**21.58**	**254, 350**		**22.00**	**254, 350**	**286**	
Prangos ferulacea								
	**1**	**18.25**	**256, 354**		**18.60**	**256, 354**	**478**	
	**2**	**20.02**	**254, 354**		**20.33**	**254, 352**	**492**	

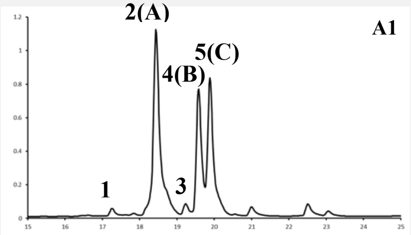

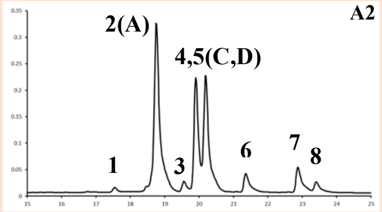

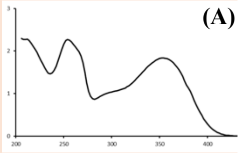

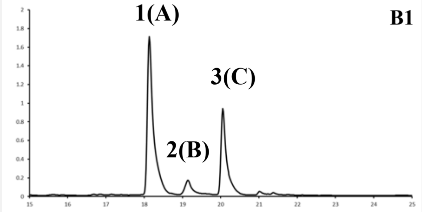

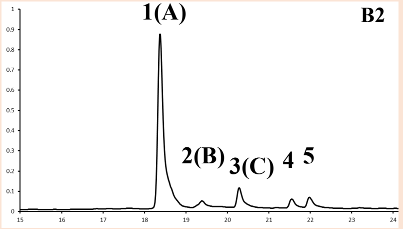

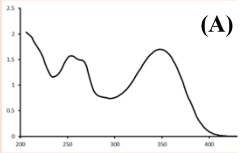

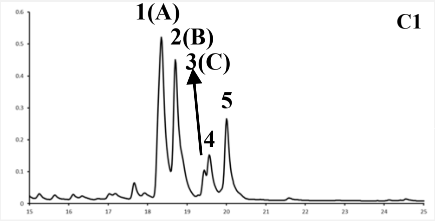

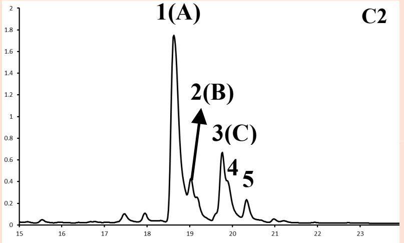

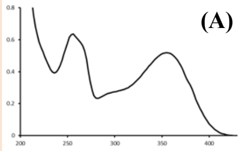

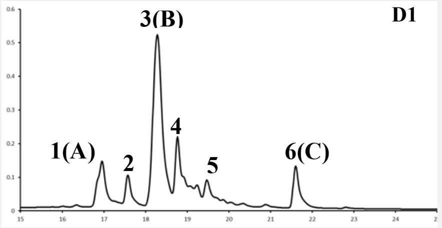

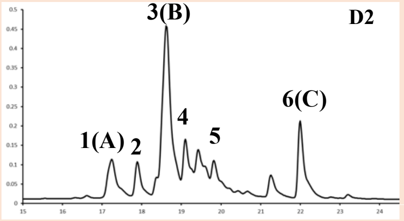

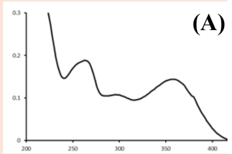

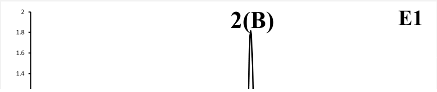

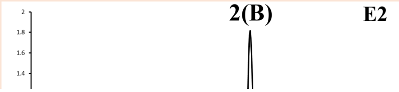

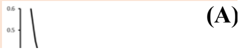

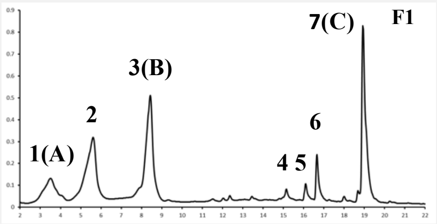	2	5.60	258, 378	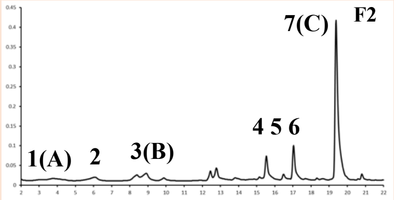	6.05	257, 377	1084	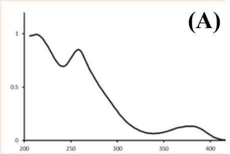
	**3**	**8.43**	**256, 378**		**8.89**	**257, 378**	**1084**	
	4	15.24	255, 363		15.51	256, 362	634	
	5	16.16	253, 378		16.45	254, 377	434	
	6	16.76	252, 359		17.01	252, 359	448	
	**7**	**18.93**	**249, 368**		**19.38**	**250, 368**	**301**	
Vitis vinifera 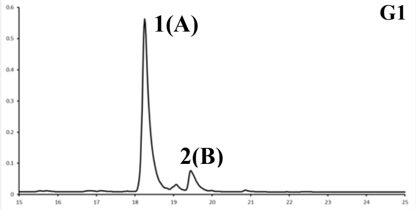	**1**	**18.25**	**256, 354**	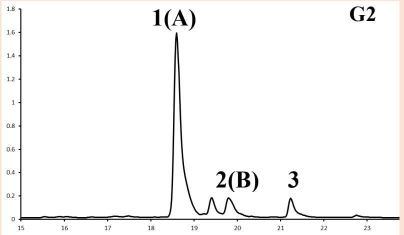	**18.60**	**256,354**	**478**	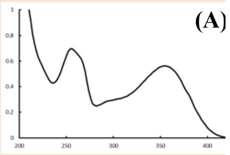
	**2**	**19.45**	**264, 348**		**19.80**	**264, 348**	**448**	
	3	-	-		21.23	254, 370	302	
