# Supplementary material for: Traditional Yellow Dyes Used in the 21st Century in Central Iran: The Knowledge of Master Dyers Revealed by HPLC-DAD and UHPLC-HRMS/MS
Source: Molecules. 2020 Feb 18;25(4):908. doi: 10.3390/molecules25040908 (PMC7070888; doi:10.3390/molecules25040908)
Supplement: Supplementary file 1 [file molecules-25-00908-s001.zip › Supplemental material/Supplementary table S3.pdf]

Table S3. Flavonoid references: common and scientific names, molecular structure, retention time, absorbance maxima and UV-VIS spectra as acquired by HPLC-DAD.

Name	Structure	t_R (min)	λ_{max} (nm)	Spectra UV-VIS
Luteolin 7-*O*-glucoside<br>*Luteolin 7-O-β-D-glucoside*	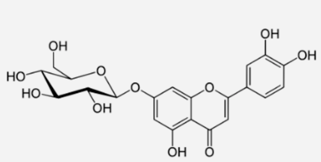	18.40	254<br>350	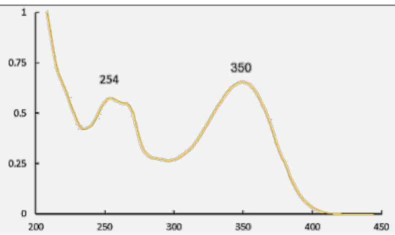
Quercetin<br>*3,3 ′, 4′, 5,7-Pentahydroxyflavone dihydrate*	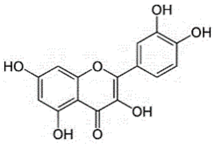	20.90	254<br>372	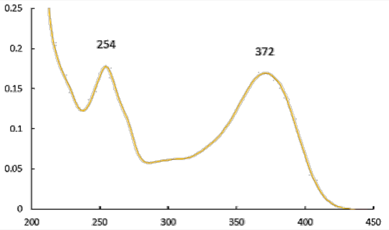
Luteolin<br>*3 ′, 4′, 5,7-Tetrahydroxyflavone*	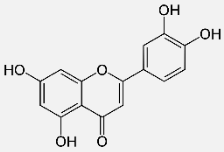	21.76	254<br>350	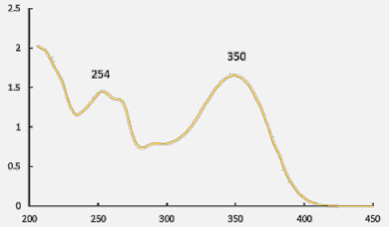
Isorhamnetin<br>*3′-Methylquercetin*	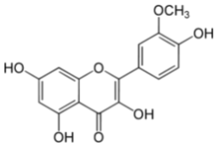	23.30	254<br>370	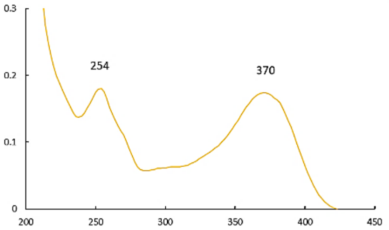
Rhamnetin<br>*7-Methoxyquercetin*	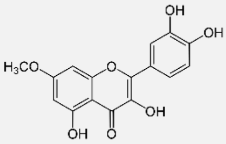	23.65	254<br>372	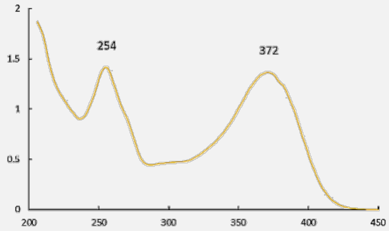
Kaempferol<br>*3,4′,5,7-tetrahydroxyflavone*	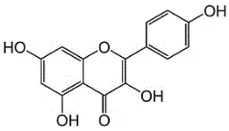	22.39	264<br>366	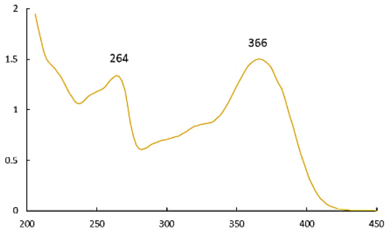
Apigenin<br>*5,7,4′-Trihydroxyflavone*	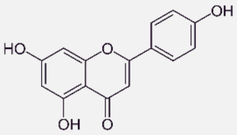	22.80	266<br>338	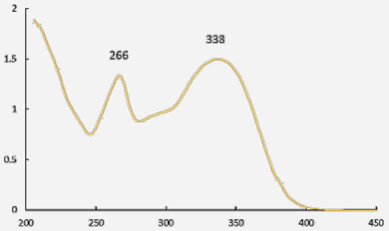

Name	Structure	t_R (min)	λ_{max} (nm)	Spectra UV-VIS
Luteolin-3',7-di-*O*-glucoside	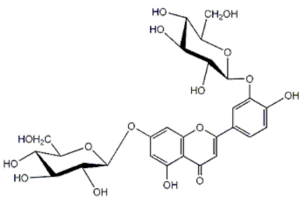	17.3	268<br>340	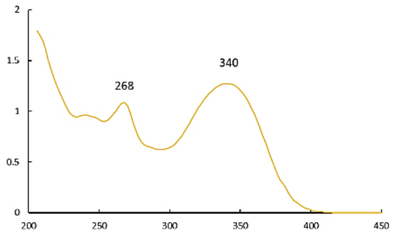
