# Supplementary figures and images for: Traditional Yellow Dyes Used in the 21st Century in Central Iran: The Knowledge of Master Dyers Revealed by HPLC-DAD and UHPLC-HRMS/MS
Source: Molecules. 2020 Feb 18;25(4):908. doi: 10.3390/molecules25040908 (PMC7070888; doi:10.3390/molecules25040908)

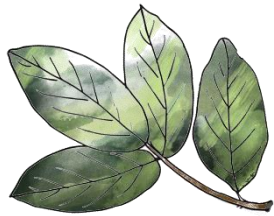

**Leaves**

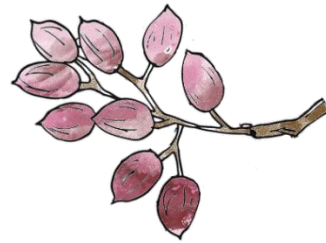

**Cluster**

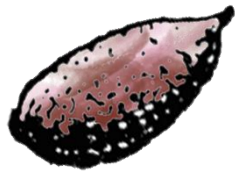

**Fleshy hull (epicarp and mesocarp)**

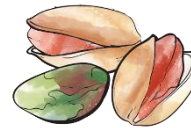

**Seed and shell (endocarp)**

Supplement: Supplementary file 1 [file molecules-25-00908-s001.zip › Supplemental material/Fig S1_Morphology of Pistacia vera.docx.pdf]
